# Genome-wide association study identifies novel loci for type 2 diabetes-attributed end-stage kidney disease in African Americans

**DOI:** 10.1186/s40246-019-0205-7

**Published:** 2019-05-15

**Authors:** Meijian Guan, Jacob M. Keaton, Latchezar Dimitrov, Pamela J. Hicks, Jianzhao Xu, Nicholette D. Palmer, Lijun Ma, Swapan K. Das, Yii-Der I. Chen, Josef Coresh, Myriam Fornage, Nora Franceschini, Holly Kramer, Carl D. Langefeld, Josyf C. Mychaleckyj, Rulan S. Parekh, Wendy S. Post, Laura J. Rasmussen-Torvik, Stephen S. Rich, Jerome I. Rotter, John R. Sedor, Denyse Thornley-Brown, Adrienne Tin, James G. Wilson, Barry I. Freedman, Donald W. Bowden, Maggie C. Y. Ng, FIND Consortium, A. Bobelu, A. Horvath, A. Pickens, B. Kessing, B. Waseta, B. I. Freedman, B. S. Kasinath, C. Garcia, C. Jefferson, C. Luethe, C. D. Langefeld, C. S. Brown, C. Winkler, D. Thornley-Brown, D. Warnock, D. J. Leehey, D. W. Bowden, E. Hernandez, E. Ipp, F. Hariri, F. Thameem, G. Barone, G. Brooks, G. Jun, G. M. Dunston, H. Abboud, I. Sili, J. Chester, J. Fondran, J. LaPage, J. Molineros, J. Rioux, J. Rotter, J. Schelling, J. Sewemaenewa, J. Tayek, J. Gonzalez, J. M. Olson, J. P. Briggs, J. R. Sedor, J. Wolford, K. Kramp, K. Taylor, K. A. B. Goddard, L. Cardon, L. Getz-Fradley, L. Humbert, L. Jones, L. Kao, L. Meoni, L. M. Phillips, M. Aguilar, M. Budgett, M. Klag, M. Pahl, M. Spainhour, M. Stern, M. Thompson, M. F. Saad, M. F. Seldin, M. Scavini, M. W. Smith, N. Arar, O. F. Kohn, P. Zager, P. L. Kimmel, R. Chakraborty, R. Duggirala, R. Hanson, R. Igo, R. Juan, R. Lovelace, R. Parekh, R. Rasooly, R. Spielman, R. Young, R. C. Elston, R. G. Nelson, R. L. Hanson, S. Adler, S. Ialacci, S. Snyder, S. Viverette, S. Warren, S. B. Nicholas, S. C. Satko, S. J. O’Brien, S. K. Iyengar, S. S. Rich, T. Hostetter, T. Whitehead, V. Shah, W. C. Knowler, X. Guo, Y.-D. I. Chen

**Affiliations:** 1 Center for Genomics and Personalized Medicine Research, Wake Forest School of Medicine, Winston-Salem, NC USA; 2 Center for Diabetes Research, Wake Forest School of Medicine, Winston-Salem, NC USA; 3 Department of Biochemistry, Wake Forest School of Medicine, Winston-Salem, NC 27157 USA; 4 Department of Internal Medicine, Section on Nephrology, Wake Forest School of Medicine, Winston-Salem, NC USA; 5 Department of Internal Medicine, Section on Endocrinology, Wake Forest School of Medicine, Winston-Salem, NC USA; 6 Institute for Translational Genomics and Population Sciences, Los Angeles Biomedical Research Institute at Harbor-UCLA Medical Center, Torrance, CA USA; 7 Department of Epidemiology, Johns Hopkins Bloomberg School of Public Health, Baltimore, MD USA; 8 Institute of Molecular Medicine, University of Texas Health Science Center at Houston, Houston, TX USA; 9 Department of Epidemiology, Gillings School of Public Health, University of North Carolina at Chapel Hill, Chapel Hill, NC USA; 10 Departments of Public Health Sciences and Medicine, Division of Nephrology and Hypertension, Loyola University Chicago, Maywood, IL USA; 11 Department of Medicine, Hines Veteran’s Affairs Medical Center, Hines, IL USA; 12 Center for Public Health Genomics, Division of Public Health Sciences, Wake Forest School of Medicine, Winston-Salem, NC USA; 13 Department of Biostatistical Sciences, Division of Public Health Sciences, Wake Forest School of Medicine, Winston-Salem, NC USA; 14 Center for Public Health Genomics, University of Virginia, Charlottesville, VA USA; 15 Departments of Paediatrics and Medicine, Hospital for Sick Children, University Health Network and the University of Toronto, Toronto, ON Canada; 16 Department of Preventive Medicine, Center for Genetic Medicine, Northwestern University Feinberg School of Medicine, Chicago, IL USA; 17 Division of Genomic Outcomes, Departments of Pediatrics and Medicine, Los Angeles Biomedical Research Institute at Harbor-UCLA Medical Center, Torrance, CA USA; 18 Molecular Medicine, Cleveland Clinic Lerner College of Medicine, Cleveland, OH USA; 19 Glickman Urology and Kidney Institute, Lerner Research Institute, Cleveland Clinic, Cleveland, OH USA; 20 Nephrology, University of Alabama Birmingham, Birmingham, AL USA; 21 Department of Physiology and Biophysics, University of Mississippi Medical Center, Jackson, MS USA

**Keywords:** African Americans, Genome-wide association study, Type 2 diabetes, Diabetic kidney disease, End-stage kidney disease

## Abstract

**Background:**

End-stage kidney disease (ESKD) is a significant public health concern disproportionately affecting African Americans (AAs). Type 2 diabetes (T2D) is the leading cause of ESKD in the USA, and efforts to uncover genetic susceptibility to diabetic kidney disease (DKD) have had limited success. A prior genome-wide association study (GWAS) in AAs with T2D-ESKD was expanded with additional AA cases and controls and genotypes imputed to the higher density 1000 Genomes reference panel. The discovery analysis included 3432 T2D-ESKD cases and 6977 non-diabetic non-nephropathy controls (*N* = 10,409), followed by a discrimination analysis in 2756 T2D non-nephropathy controls to exclude T2D-associated variants.

**Results:**

Six independent variants located in or near *RND3/RBM43*, *SLITRK3*, *ENPP7*, *GNG7*, and *APOL1* achieved genome-wide significant association (*P* < 5 × 10^−8^) with T2D-ESKD. Following extension analyses in 1910 non-diabetic ESKD cases and 908 non-diabetic non-nephropathy controls, a meta-analysis of 5342 AA all-cause ESKD cases and 6977 AA non-diabetic non-nephropathy controls revealed an additional novel all-cause ESKD locus at *EFNB2* (rs77113398; *P* = 9.84 × 10^–9^; OR = 1.94). Exclusion of *APOL1* renal-risk genotype carriers identified two additional genome-wide significant T2D-ESKD-associated loci at *GRAMD3* and *MGAT4C*. A second variant at *GNG7* (rs373971520; *P* = 2.17 × 10^–8^, OR = 1.46) remained associated with all-cause ESKD in the *APOL1*-negative analysis.

**Conclusions:**

Findings provide further evidence for genetic factors associated with advanced kidney disease in AAs with T2D.

**Electronic supplementary material:**

The online version of this article (10.1186/s40246-019-0205-7) contains supplementary material, which is available to authorized users.

## Introduction

Increasing evidence suggests that genetic factors play a major role in susceptibility to end-stage kidney disease (ESKD). This is particularly relevant in African Americans (AAs) where incidence rates of ESKD are more than threefold greater than European Americans (EAs) [1]. The mortality rates for ESKD, dialysis, and transplant patients are 136, 166, and 30 per 1000 patient-years, respectively, and account for 7.2% of Medicare-paid claims costs [1]. Diabetes, of which 95% of patients have type 2 diabetes (T2D), remains the leading reported cause of ESKD in the USA accounting for > 44% of cases [1]. Improvements in glycemic, lipid, and blood pressure control have not markedly reduced the prevalence of diabetic kidney disease (DKD) [1, 2]. In addition, familial aggregation of DKD is independent from socioeconomic status and established environmental risk factors [3, 4]. Although the G1 and G2 alleles in the apolipoprotein L1 gene (*APOL1*) contribute to 50–70% of non-diabetic ESKD in AAs, they do not fully explain the excess risk of T2D-attributed ESKD (T2D-ESKD) in this population [5–7].

**Fig. 1:**
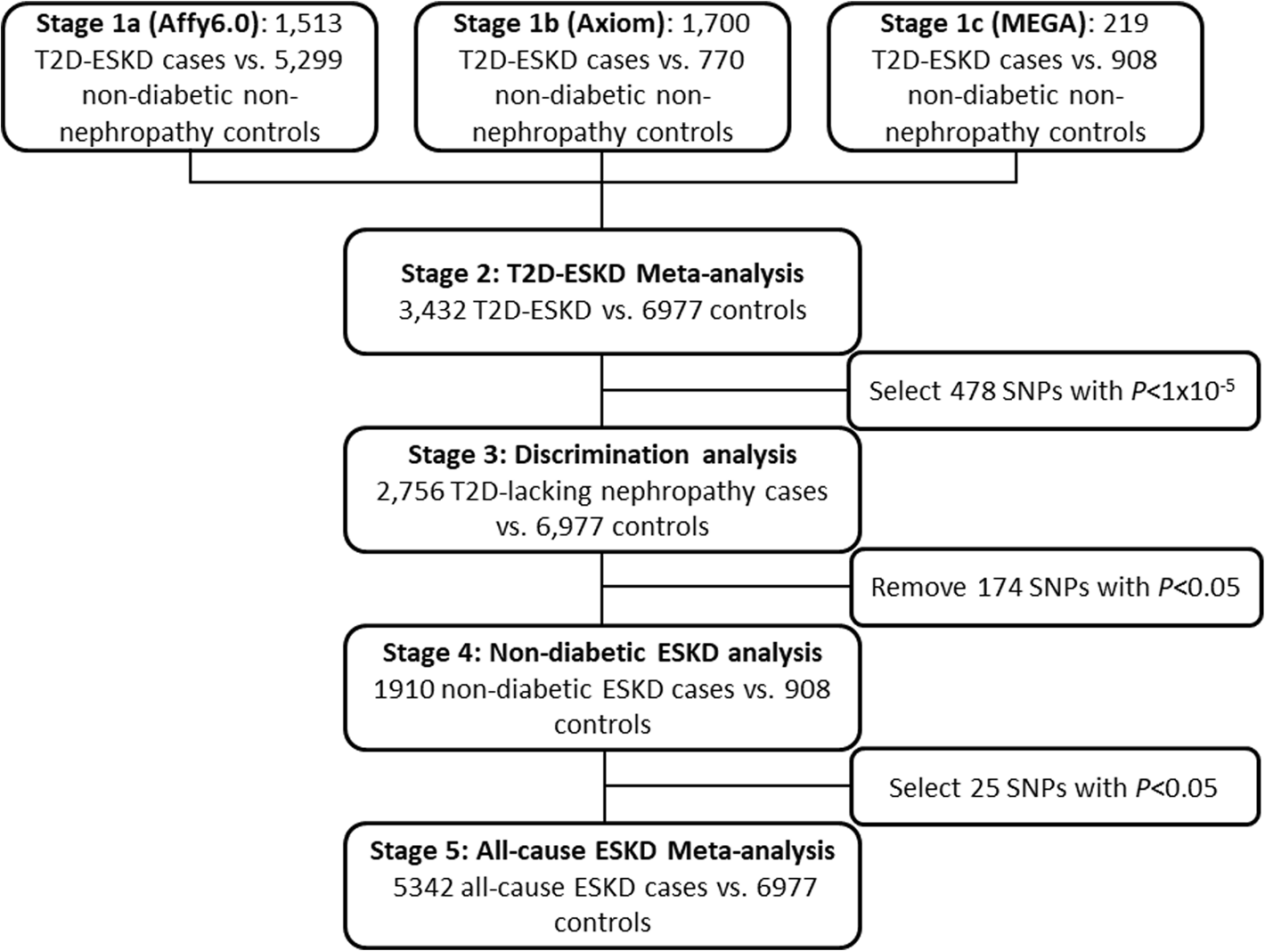
Workflow of T2D-ESKD GWAS in AAs (baseline model)

Genome-wide association studies (GWAS) have identified > 70 genome-wide significant variants associated with chronic kidney disease (CKD), albuminuria, or renal function in European ancestry populations [8–11]. However, few loci were associated with DKD in diverse populations and they do not consistently replicate, in part due to limited sample sizes [12–18]. The etiology of kidney complications in patients with T2D is likely more heterogeneous than in patients with type 1 diabetes [16]. Therefore, careful phenotyping and larger sample sizes are required to improve statistical power. To explore the genetic architecture of advanced kidney disease in T2D, we extended our previous GWAS (2890 ESKD patients and 1719 non-diabetic non-nephropathy controls) efforts in a larger sample of AAs with severe kidney disease. Association analyses were performed in six independent AA cohorts (Wake Forest School of Medicine, WFSM; Family Investigation of Nephropathy and Diabetes, FIND; Atherosclerosis Risk in Communities Study, ARIC; Multi-Ethnic Study of Atherosclerosis, MESA; Jackson Heart Study, JHS; and Coronary Artery Risk Development in Young Adults, CARDIA) for T2D-ESKD or non-diabetic ESKD through a multi-stage study design (Fig. 1). This encompassed 15,075 AAs classified into four phenotypic groups: T2D-ESKD cases (*N* = 3432), non-diabetic non-nephropathy controls (*N* = 6977), T2D-lacking nephropathy controls (*N* = 2756), and non-diabetic ESKD cases (*N* = 1910).

In the discovery stage, GWAS was performed in 3432 T2D-ESKD cases and 6977 non-diabetic non-nephropathy controls, followed by a discrimination analysis to exclude T2D-associated loci in 2756 T2D non-nephropathy controls. Extension analyses were performed in 1910 AAs with non-diabetic ESKD and 908 non-nephropathy controls to assess the contribution of T2D-ESKD-associated loci to non-diabetic kidney disease. A meta-analysis of diabetic and non-diabetic ESKD cases assessed genetic associations in all-cause ESKD. APOL1-associated forms of non-diabetic kidney disease and T2D often co-exist in patients. As such, many diabetic patients with kidney disease may be misclassified as having DKD, since diagnostic kidney biopsies are usually not performed. Herein, a second GWAS analysis excluding individuals with *APOL1* renal-risk genotypes was performed to minimize misclassification of T2D-ESKD.

## Results

### Study overview

This study has > 80% power to detect common variants (MAF ≥ 0.10) with moderate effect (OR ≥ 1.3) at a significance level of 5 × 10^−8^ (http://csg.sph.umich.edu/abecasis/cats/). In all, seven genome-wide significant loci (*P* < 5 × 10^−8^) associated with T2D-ESKD were identified in either the baseline model (*RND3/RBM43*, *SLITRK3*, *ENPP7*, *GNG7*, and *APOL1*) or *APOL1*-negative model (*ENPP7*, *GRAMD3*, and *MGAT4C*). In addition to *APOL1*, two loci, *EFNB2* and *GNG7*, also reached genome-wide significance in the all-cause ESKD meta-analysis under either the baseline or *APOL1*-negative models.

### Clinical characteristics of study participants

Tables 1 and 2 include detailed characteristics of study participants. ESKD cases were recruited from the WFSM (Affy6.0, Axiom, and MEGA), FIND, and ARIC studies. Individuals with T2D-ESKD or T2D-lacking nephropathy were older (or of similar age) compared to the non-diabetic non-nephropathy controls at recruitment. However, the average age at diagnosis of T2D in T2D-ESKD cases and T2D-lacking nephropathy controls were younger than non-diabetic non-nephropathy controls at recruitment. All T2D non-nephropathy controls and non-diabetic, non-nephropathy controls had normal eGFR ≥ 60 ml/min/1.73m^2^. In addition, non-diabetic non-nephropathy controls had fasting glucose levels < 126 mg/dl. Controls with T2D-lacking nephropathy were more obese than T2D-ESKD or non-diabetic ESKD cases and non-diabetic, non-nephropathy controls, except that T2D-ESKD cases in ARIC were more obese than the other groups.

**Table 1:** Clinical characteristics of participants from the Affy6.0 datasets (stage 1a)

	WFSM		FIND		ARIC			JHS		MESA		CARDIA	
	T2D-ESKD	Non-diabetic non-nephropathy	T2D-ESKD	T2D-lacking nephropathy	T2D-ESKD	Non-diabetic non-nephropathy	T2D-lacking nephropathy	Non-diabetic non-nephropathy	T2D-lacking nephropathy	Non-diabetic non-nephropathy	T2D-lacking nephropathy	Non-diabetic non-nephropathy	T2D-lacking nephropathy
*N*	790	891	627	299	96	1318	807	1569	343	774	278	747	165
Females (%)	63	56	54	76	65	62	63	59	68	54	53	60	66
Age (years)	62.1 ± 10.0	47.2 ± 11.8	56.84 ± 12.0	59.8 ± 11.1	68.2 ± 6.6	60.2 ± 6.1	60.3 ± 5.8	53.4 ± 11.6	59.3 ± 9.1	63.9 ± 9.2	65.6 ± 9.2	47.6 ± 5.0	49.4 ± 4.3
Age of onset of diabetes (years)	40.4 ± 11.8	–	–	–	47.3 ± 10.4	–	57.4 ± 10.8	-	44.9 ± 11.3	–	–	–	32.3 ± 7.9
Duration of diabetes prior to ESKD (years)	18.5 ± 9.6	–	–	–	20.5 ± 8.6	–	–	–	–	–	–	–	–
Duration of ESKD (years)	3.3 ± 3.6	–	–	–	0.6 ± 1.8	–	–	–	–	–	–	–	–
Fasting serum glucose (mg/dl)	193 ± 64	–	–	–	293 ± 115	105 ± 9	192 ± 94	90 ± 8	156 ± 66	98 ± 10	164 ± 59	96 ± 9	167 ± 66
eGFR (ml/min/1.73m^2^)	-	96.7 ± 21.3	–	–	-	78.6 ± 12.5	79.5 ± 12.6	95.5 ± 17.6	92.7 ± 18.5	78.9 ± 12.1	82.9 ± 16.0	91.1 ± 13.1	95.6 ± 13.6
Body mass index (kg/m^2^)	29.8 ± 7.0	30.0 ± 7.0	–	–	33.4 ± 7.3	29.2 ± 6.1	32.2 ± 6.8	31.6 ± 7.7	35.4 ± 7.7	29.8 ± 5.8	31.8 ± 6.5	–	–

Categorical data expressed as percentage; continuous data as mean ± SD

*Abbreviations*: *T2D* type 2 diabetes, *ESKD* end-stage kidney disease, *N* number, *eGFR* estimated glomerular filtration rate

**Table 2:** Clinical characteristics of participants genotyped using Axiom and MEGA arrays (stage 1b and 1c)

	WFSM-Axiom			WFSM-MEGA			
	T2D-ESKD	Non-diabetic non-nephropathy	T2D-lacking nephropathy	T2D-ESKD	Non-diabetic non-nephropathy	T2D-lacking nephropathy	Non-diabetic ESKD
*N*	1700	770	663	219	908	201	1910
Females (%)	56	49	64	49	59	62	41
Age (years)	62.0 ± 10.8	47.9 ± 12.0	55.7 ± 11.6	62.0 ± 11.0	44.8 ± 13.9	55.8 ± 9.5	55.4 ± 14.4
Age of onset of diabetes (years)	39.7 ± 12.8	–	46.2 ± 12.3	37.8 ± 9.6	–	43.7 ± 10.3	–
Duration of diabetes prior to ESKD (years)	19.1 ± 10.0	–	–	20.4 ± 9.5	–	–	–
Duration of ESKD (years)	3.6 ± 3.6	–	–	4.1 ± 3.1	–	–	6.2 ± 5.8
Fasting serum glucose (mg/dl)	184 ± 89	96 ± 22	163 ± 92	127 ± 33	96 ± 11	174 ± 62	90 ± 3
eGFR (ml/min/1.73m^2^)	–	96.0 ± 20.8	91.3 ± 19.7	–	85.9 ± 17.3	95.2 ± 17.3	–
Body mass index (kg/m^2^)	30.7 ± 7.1	29.6 ± 7.4	33.1 ± 7.8	30.8 ± 7.0	29.7 ± 6.6	33.0 ± 6.5	27.8 ± 7.2

Categorical data expressed as percentage; continuous data as mean ± SD

*Abbreviations*: *T2D* type 2 diabetes, *ESKD* end-stage kidney disease, *N* number, *eGFR* estimated glomerular filtration rate

### Stage 1 and stage 2 T2D-ESKD association analysis

In stage 1 discovery, GWAS was conducted separately in three datasets: (1) 1513 T2D-ESKD cases and 5299 non-diabetic non-nephropathy controls genotyped on Affy6.0, contributed by WFSM, FIND, ARIC, JHS, MESA, and CARDIA (stage 1a); (2) 1700 T2D-ESKD cases and 770 non-diabetic non-nephropathy controls from WFSM genotyped with Axiom Biobank genotyping array (stage 1b); and (3) 219 T2D-ESKD cases and 908 non-diabetic, non-nephropathy controls from WFSM genotyped on MEGA (stage 1c). A meta-analysis (stage 2) was performed to combine association results for 3432 T2D-ESKD cases and 6977 non-diabetic, non-nephropathy controls from stages 1a, 1b, and 1c. An inflation factor *λ* of 1.013 was observed after correcting for genomic control (Additional file 1: Figure S1), suggesting that population structure and cryptic relatedness were sufficiently adjusted. Among variants demonstrating suggestive associations (*P* < 1 × 10^−5^), 59 variants with *I*^2^ ≥ 80% were excluded due to high heterogeneity in effect sizes across studies. A total of 478 remaining variants were assessed in a discrimination analysis (81 of them achieved genome-wide significance; Additional file 1: Table S2).

### Stage 3 discrimination analysis

**Fig. 2:**
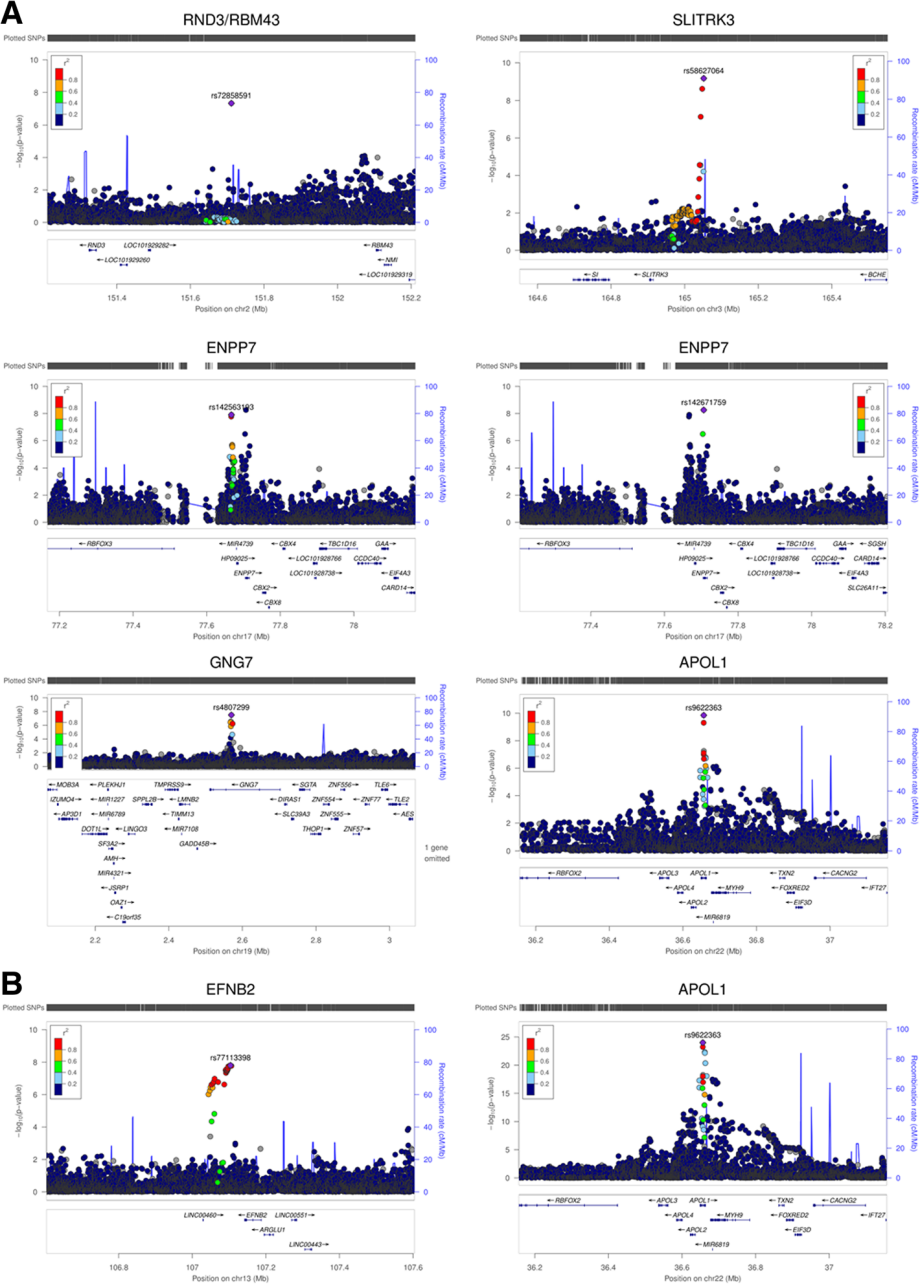
Locus plots of genome-wide associations in the baseline model. **a** Locus plots of T2D-ESKD associations at *P* < 5 × 10^−8^ in the baseline model. **b** Locus plots of all-cause ESKD-associated variants at *P* < 5 × 10^− 8^ in the baseline model. Abbreviations: T2D, type 2 diabetes; ESKD, end-stage kidney disease; Baseline model: adjusted for age, sex, and PC1. *APOL1* risk genotype carriers were included; reference genome: hg19/1000 Genomes Nov 2014 AFR.

To determine whether the T2D-ESKD associations identified in stage 1 meta-analysis were driven by association with T2D per se, a discrimination analysis was performed for T2D contrasting 2756 AAs with T2D-lacking nephropathy with 6977 non-diabetic non-nephropathy controls from stage 1 (Affy6.0, Axiom, MEGA; Additional file 1: Table S3). We subsequently excluded 174 of 478 T2D-ESKD-associated variants nominally associated with T2D in the absence of nephropathy. Among the remaining T2D-ESKD associations, top variants representing 6 independent associations achieved genome-wide significance (Table 3, Fig. 2a). The strongest association was observed for rs9622363 located at *APOL1* (*P* = 1.42 × 10^−10^, OR = 0.77, EAF = 0.45). This variant was in moderate linkage disequilibrium (*r*^2^ = 0.33 and 0.34, respectively in YRI) with the *APOL1* G1 alleles (rs60910145, rs73885319) associated with non-diabetic ESKD [6]. The second strongest association was at rs58627064, an intergenic variant located near *SLITRK3*, (*P* = 6.81 × 10^−10^, OR = 1.62, EAF = 0.06). Two independent signals, rs142563193 (*P* = 1.24 × 10^−8^, OR = 0.74, EAF = 0.23) and rs142671759 (*P* = 5.53 × 10^−9^, OR = 2.26, EAF = 0.02) on chromosome 17 located near *ENPP7*, respectively, were also genome-wide significant. In addition, two associations with T2D-ESKD, rs4807299 (*P* = 3.21 × 10^−8^, OR = 1.67, EAF = 0.05) located in *GNG7* and rs72858591 (*P* = 4.54 × 10^−8^, OR = 1.43, EAF = 0.10) located in *RND3/RBM43* were identified (Fig. 1a).

**Table 3:** Genome-wide significant T2D-ESKD associations (*P* < 5 × 10^−8^) in baseline model

Lead variant	CHR	POS	Locus	Effect/other alleles	Stage 1a (1513 T2D-ESKD cases vs. 5299 non-diabetic non-nephropathy controls)				Stage 1b (1700 T2D-ESKD cases vs. 770 non-diabetic non-nephropathy controls)				Stage 1c (219 T2D-ESKD cases vs. 908 non-diabetic non-nephropathy controls)				Stage 2: Meta-analysis (3432 T2D-ESKD cases vs. 6977 non-diabetic non-nephropathy controls)			
					EAF	OR (95% CI)	*P*	Info	EAF	OR (95% CI)	*P*	Info	EAF	OR (95% CI)	*P*	Info	EAF	OR (95% CI)	*P*	HetP
rs72858591	2	151711452	*RND3/RBM43*	C/T	0.10	1.55 (1.33,1.8)	1.32E−08	0.96	0.09	1.19 (0.92,1.54)	0.18	0.98	0.10	1.14 (0.72,1.82)	0.57	0.97	0.10	1.43 (1.26,1.62)	4.54E−08	0.15
rs58627064	3	165051826	*SLITRK3*	T/G	0.07	1.84 (1.53,2.2)	4.38E−11	0.91	0.06	1.19 (0.87,1.64)	0.27	0.97	0.06	1.25 (0.7,2.23)	0.46	1.00	0.06	1.62 (1.39,1.89)	6.81E−10	0.045
rs142563193	17	77667171	*ENPP7*	A/G	0.22	0.69 (0.6,0.78)	3.27E−08	0.64	0.23	0.81 (0.68,0.96)	0.018	0.97	0.25	0.91 (0.66,1.26)	0.57	0.97	0.23	0.74 (0.67,0.82)	1.24E−08	0.15
rs142671759	17	77706698	*ENPP7*	C/T	0.03	2.82 (2.05,3.87)	1.53E−10	0.73	0.02	1.15 (0.62,2.12)	0.66	0.82	0.02	1.53 (0.57,4.13)	0.40	0.92	0.02	2.26 (1.72,2.97)	5.53E−09	0.029
rs4807299	19	2570002	*GNG7*	A/C	0.05	1.95 (1.58,2.41)	7.31E−10	0.79	0.04	1.12 (0.76,1.65)	0.56	0.88	0.05	1.16 (0.58,2.32)	0.68	0.92	0.05	1.67 (1.4,2)	3.21E−08	0.028
rs9622363	22	36656555	*APOL1*	A/G	0.46	0.78 (0.7,0.85)	2.55E−07	0.87	0.44	0.77 (0.66,0.89)	0.00040	1.00	0.48	0.77 (0.58,1.03)	0.08	0.97	0.45	0.77 (0.72,0.84)	1.42E−10	0.995

Baseline model: adjusted for age, sex, and PC1, *APOL1* risk genotype carriers were included

*Abbreviations*: *T2D* type 2 diabetes, *ESKD* end-stage kidney disease, *CHR* chromosome, *POS* position, *EAF* effect allele frequency, *OR* odds ratio, *CI* confidence interval, *P P* value, *Info* imputation quality, *HetP* heterogeneity *P* value

### Stage 4 non-diabetic ESKD analysis and stage 5 all-cause ESKD meta-analysis

After the discrimination stage, 304 variants exhibiting suggestive association with T2D-ESKD (*P* < 1 × 10^−5^) were tested in 1910 independent non-diabetic ESKD cases and 908 controls from stage 1c. The goal of the stage 4 analysis was to evaluate the contribution of T2D-ESKD-associated loci to non-diabetic kidney disease. After excluding variants with heterogeneity *I*^2^ ≥ 80%, 25 variants were nominally associated with non-diabetic ESKD (*P* < 0.05). Strong associations (1.27 × 10^−29^ < *P* < 8.86 × 10^−15^) were observed in the *APOL1-MYH9* region, confirming their role in non-diabetic kidney disease. An all-cause ESKD meta-analysis, including 5342 all-cause ESKD and 6977 non-diabetic non-nephropathy controls, was conducted to evaluate the generalizability of the 25 T2D-ESKD-associated variants with broader forms of ESKD (stage 5). Thirty-five genome-wide significant variants at two loci were found to be associated with all-cause ESKD, including 15 variants within or near *EFNB2* and 20 variants at *APOL1* (Fig. 1b). The top association in *APOL1* was rs9622363 (*P* = 1.96 × 10^−25^, OR = 0.68, EAF = 0.43), and the top signal near *EFNB2* was rs77113398 (*P* = 9.84 × 10^−9^, OR = 1.94, EAF = 0.023) (Table 4, Fig. 2b). Four additional independent loci demonstrated suggestive association (*P* < 5 × 10^−6^) with all-cause ESKD at *LPP*, *FSTL5*, *OPRK1/ATPV1H*, and *SYBU/KCNV1* (Table 4, Fig. 2b).

**Table 4:** All-cause ESKD-associated variants at *P* < 5 × 10^–6^ in baseline model

Lead variant	CHR	POS	Locus	Effect/other alleles	Info	Stage 2 (3432 T2D-ESKD cases vs. 6977 non-diabetic non-nephropathy controls)			Stage 4 (1910 non-diabetic ESKD cases vs. 908 non-diabetic non-nephropathy controls)			Stage 5: Meta-analysis (5342 all-cause ESKD cases vs. 6977 non-diabetic non-nephropathy controls)			
						EAF	OR (95% CI)	*P*	EAF	OR (95% CI)	*P*	EAF	OR (95% CI)	*P*	HetP
rs76971802	3	188607071	*LPP*	T/C	0.95	0.087	1.35 (1.19,1.54)	8.66E−06	0.084	1.42 (1.08,1.87)	0.013	0.087	1.35 (1.2,1.52)	1.36E−06	0.86
rs5863506	4	162909217	*FSTL5*	TA/T	0.96	0.33	1.22 (1.12,1.32)	1.64E−06	0.33	1.19 (1.01,1.40)	0.033	0.33	1.21 (1.12,1.30)	4.57E−07	0.87
rs141746998	8	54310938	*OPRK1/ATP6V1H*	CAT/C	0.94	0.031	0.62 (0.5,0.76)	9.40E−06	0.034	0.61 (0.39,0.93)	0.022	0.031	0.61 (0.5,0.75)	1.44E−06	0.96
rs11997465	8	110891977	*SYBU/KCNV1*	C/G	0.99	0.28	1.22 (1.12,1.33)	3.21E−06	0.28	1.21 (1.02,1.43)	0.026	0.28	1.22 (1.13,1.31)	6.72E−07	0.65
rs77113398	13	107103906	*EFNB2*	A/G	0.93	0.023	1.94 (1.52,2.47)	1.25E−07	0.021	1.87 (1.08,3.24)	0.025	0.023	1.94 (1.55,2.43)	9.84E−09	0.96
rs9622363	22	36656555	*APOL1*	A/G	0.95	0.45	0.77 (0.72,0.84)	1.42E−10	0.35	0.42 (0.36,0.48)	4.32E−29	0.43	0.68 (0.64,0.73)	1.96E−25	1.14E−09

Baseline model: adjusted for age, sex, and PC1; *APOL1* risk genotype carriers were included

*Abbreviations*: *T2D* type 2 diabetes, *ESKD* end-stage kidney disease, *CHR* chromosome, *POS* position, *EAF* effect allele frequency, *OR* odds ratio, *CI* confidence interval, *P*, *P* value, *Info* average imputation quality, *HetP* heterogeneity *P* value

### Association analysis excluding *APOL1* renal-risk-genotype carriers

**Fig. 3:**
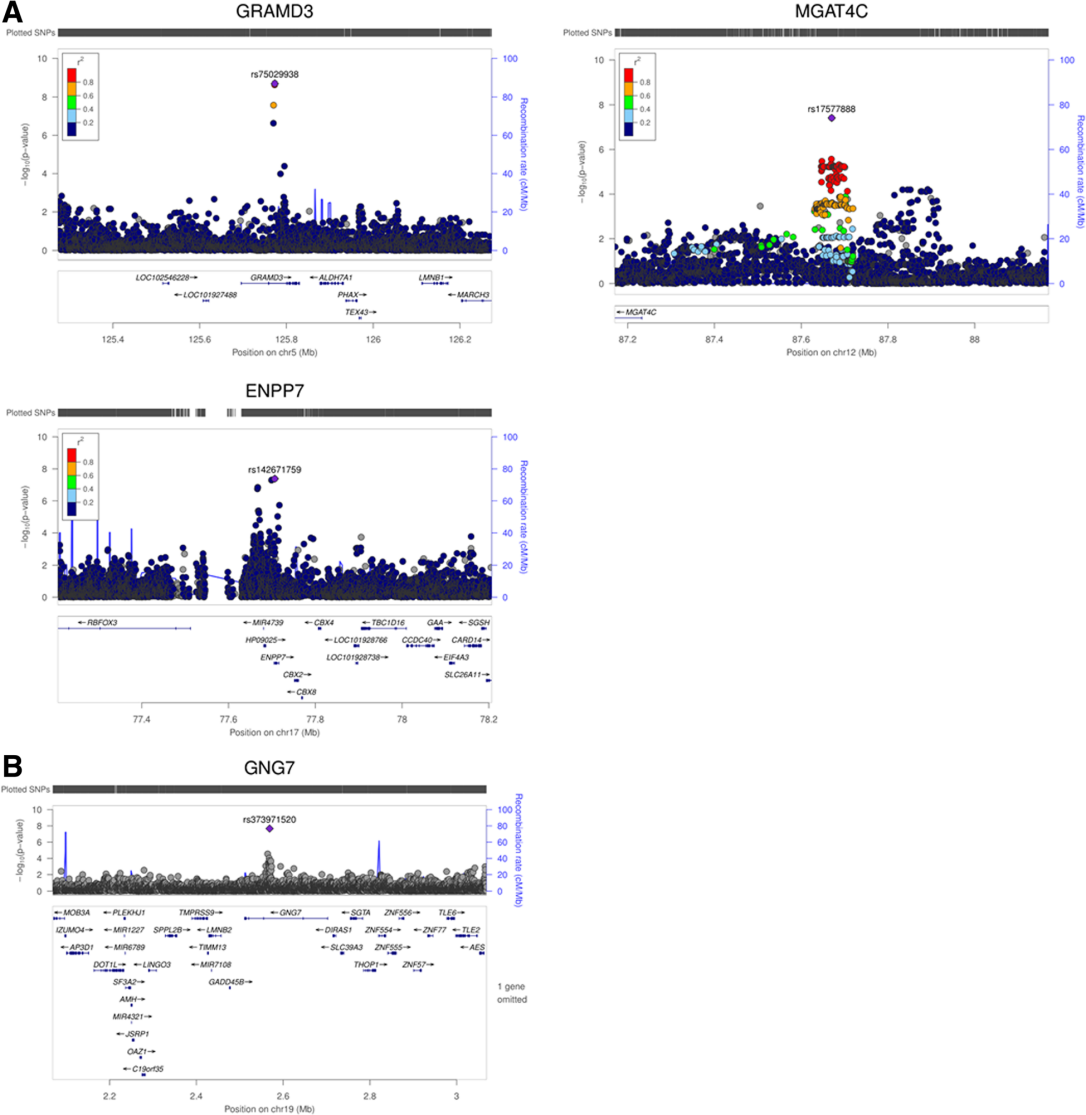
Locus plots of genome-wide associations in the *APOL1*-negative model. **a** Locus plots of T2D-ESKD associations at *P* < 5 × 10^−8^ in the *APOL1*-negative model. **b** Locus plots of all-cause ESKD associations at *P* < 5 × 10^−8^ in the *APOL1*-negative model. Abbreviations: T2D, type 2 diabetes; ESKD, end-stage kidney disease; P, *P* value; *APOL1*-negative mode, adjusted for age, sex, and PC1. *APOL1* risk genotype carriers excluded; reference genome: hg19/1000 Genomes Nov 2014 AFR.

A secondary analysis was performed excluding *APOL1* renal-risk-genotype carriers in T2D-ESKD cases and non-diabetic non-nephropathy controls (*APOL1*-negative model) to enrich for T2D-associated ESKD. The baseline model showed a strong association of *APOL1* and *MYH9* with T2D-ESKD (Table 3, Fig. 1a) suggesting that some cases may have been misclassified and more likely had non-diabetic ESKD. A total of 664 T2D-ESKD cases and 918 non-diabetic non-nephropathy controls were excluded from stage 1 analysis, leaving 2768 T2D-ESKD cases and 6059 controls in the *APOL1*-negative model. Nominal associations with T2D-ESKD (*P* < 1 × 10^−5^) were observed with 522 variants (66 of them achieved genome-wide significance; Additional file 1: Table S2), and these were selected for stage 3 discrimination analysis. Two hundred twenty-three variants that had evidence of association with T2D per se (Additional file 1: Table S4) and 24 variants showing strong heterogeneity (*I*^2^ ≥ 80) in the meta-analysis were removed. Among the genome-wide significant variants identified in the baseline model, rs142671759 in *ENPP7* (*P* = 4.10 × 10^−8^, OR = 2.30, EAF = 0.024) showed a consistent association with T2D-ESKD in the *APOL1*-negative model. Two additional variants reached genome-wide significance, rs75029938 in *GRAMD3* (*P* = 2.02 × 10^–9^, OR = 1.89, EAF = 0.042) and rs17577888 in the *MGAT4C* region (*P* = 3.87 × 10^−8^, OR = 0.67, EAF = 0.087) (Table 5, Fig. 3a).

**Table 5:** Genome-wide significant T2D-ESKD associations (*P* < 5 × 10^−8^) in *APOL1*-negative model

Lead variant	CHR	POS	Locus	Annotation	Effect/other allele	Stage 1a (1205 T2D-ESKD cases vs. 4669 non-diabetic non-nephropathy controls)				Stage 1b (1377 T2D-ESKD cases vs. 657 non-diabetic non-nephropathy controls)				Stage 1c (186 T2D-ESKD cases vs. 733 non-diabetic non-nephropathy controls)				Stage 2: Meta-analysis (2768 T2D-ESKD cases vs. 6059 non-diabetic non-nephropathy controls)			
						EAF	OR (95% CI)	*P*	Info	EAF	OR (95% CI)	*P*	Info	EAF	OR (95% CI)	*P*	Info	EAF	OR (95% CI)	*P*	HetP
rs75029938	5	125773333	*GRAMD3*	Intron	T/C	0.044	2.27 (1.78,2.88)	1.88E−11	0.87	0.036	1.01 (0.62,1.65)	0.97	0.69	0.032	1.23 (0.51,2.96)	0.65	0.94	0.042	1.89 (1.54, 2.34)	2.02E−09	0.0094
rs17577888	12	87670213	*MGAT4C*	Intergenic	T/G	0.089	0.69 (0.58,0.82)	1.99E−05	1	0.086	0.59 (0.44,0.78)	0.00029	0.99	0.073	0.84 (0.46,1.55)	0.59	0.99	0.087	0.67 (0.58, 0.77)	3.87E−08	0.50
rs142671759	17	77706698	*ENPP7*	Intron	C/T	0.025	2.71 (1.92,3.82)	1.46E−08	0.73	0.019	1.52 (0.78,2.94)	0.22	0.82	0.016	1.19 (0.39,3.7)	0.76	0.92	0.024	2.29 (1.7, 3.07)	4.1E−08	0.16

*APOL1*-negative model: adjusted for age, sex, and PC1; *APOL1* risk genotype carriers were excluded

*Abbreviations*: *T2D* type 2 diabetes, *ESKD* end-stage kidney disease, *CHR* chromosome, *POS* position, *N* number, *EAF* effect allele frequency, *OR* odds ratio, *CI* confidence interval, *P P* value, *Info* imputation quality, *HetP* heterogeneity *P* value

We further tested 275 suggestive T2D-ESKD associations that passed discrimination and had *I*^2^ < 80 in 1019 additional AA non-diabetic ESKD cases that excluded *APOL1* renal-risk-genotype carriers. Fifteen variants showed nominal evidence of association with non-diabetic ESKD. These were subsequently tested in an all-cause ESKD meta-analysis including 3787 all-cause ESKD cases and 6059 non-diabetic non-nephropathy controls of which *APOL1* renal-risk-genotype carriers were excluded. A 2-base pair deletion in *GNG7*, rs373971520 (*P* = 2.17 × 10^−8^, OR = 1.46, EAF = 0.11), achieved genome-wide significant association with all-cause ESKD (Fig. 3b). Seven additional loci displayed nominal association with all-cause ESKD (*P* < 5 × 10^−6^), including *LPP*, *ALK/YPEL5*, *MNX1-AS1/UBE3C*, *NUP98*, *LINC01075/LINC00448*, *TMCO5A*, and *SULF2/LINC01522* (Table 6). The top associations from the baseline model had moderate attenuation in significance, despite the similar effect sizes, due in part to the reduced sample size (Additional file 1: Table S5).

**Table 6:** All-cause ESKD-associated variants at *P* < 5 × 10^−6^ in *APOL1*-negative model

Lead variant	CHR	POS	Locus	Effect/other allele	*Info	Stage 2: Meta-analysis (2768 T2D-ESKD cases vs. 6059 non-diabetic non-nephropathy controls)			Stage 4 (1019 non-diabetic ESKD cases vs. 733 non-diabetic non-nephropathy controls)			Stage 5 (3787 all-cause ESKD cases vs. 6059 non-diabetic non-nephropathy controls)			
						EAF	OR (95% CI)	*P*	EAF	OR(95% CI)	*P*	EAF	OR (95% CI)	*P*	HetP
rs12472637	2	30304514	*ALK/YPEL5*	A/G	0.94	0.33	0.81 (0.74, 0.89)	6.05E−06	0.36	0.8 (0.66, 0.96)	0.019	0.33	0.82 (0.76, 0.89)	4.47E−06	0.83
rs76971802	3	188607071	*LPP*	T/C	0.95	0.09	1.43 (1.24, 1.65)	1.06E−06	0.08	1.45 (1.03, 2.05)	0.032	0.088	1.42 (1.24, 1.62)	5.76E−07	0.93
rs6459733	7	156930550	*MNX1-AS1/UBE3C*	G/C	0.97	0.39	1.25 (1.15, 0.87)	1.32E−07	0.39	1.25 (1.03, 1.53)	0.026	0.39	1.24 (1.15, 1.35)	7.17E−08	0.83
rs4910809	11	3813850	*NUP98*	C/G	0.98	0.02	0.53 (0.4, 0.7)	9.46E−06	0.02	0.53 (0.29, 0.95)	0.034	0.023	0.53 (0.41, 0.69)	2.12E−06	0.63
rs219020	13	63013622	*LINC01075/LINC00448*	C/T	0.97	0.14	1.31 (1.17, 0.86)	6.84E−06	0.11	1.38 (1.02, 1.87)	0.037	0.14	1.31 (1.17, 1.47)	2.12E−06	0.58
rs113452507	15	37954309	*TMCO5A*	C/G	0.92	0.16	1.29 (1.15, 1.45)	9.71E−06	0.16	1.38 (1.07, 1.77)	0.014	0.16	1.31 (1.18, 1.45)	8.48E−07	0.93
rs373971520	19	2568805	*GNG7*	–/CA	0.81	0.11	1.47 (1.28, 1.7)	8.97E−08	0.11	1.39 (1.01, 1.91)	0.044	0.11	1.46 (1.28, 1.67)	2.17E−08	0.016
rs6094913	20	46561443	*SULF2/LINC01522*	G/A	0.93	0.09	0.72 (0.63, 1.6)	8.28E−06	0.10	0.67 (0.48, 0.91)	0.012	0.092	0.72 (0.63, 0.83)	2.5E−06	0.70

*Abbreviations*: *T2D* type 2 diabetes, *ESKD* end-stage kidney disease, *CHR* chromosome, *POS* position, *EAF* effect allele frequency, *OR* odds ratio, *CI* confidence interval, *P P* value, *Info imputation quality, *HetP* heterogeneity *P* value;

*APOL1*-negative model: adjusted for age, sex, and PC1; *APOL1* risk genotype carriers were excluded

In addition, a third GWAS was performed with *APOL1* included as a covariate in the model (*APOL1*-adjusted model) and comparing −log (*P*) values with baseline and *APOL1*-negative models. High correlation (Person correlation coefficient *r* = 0.95) was observed between *APOL1*-adjusted and *APOL1*-negative models. The results of all three comparisons are included in the supplementary documentation (Additional file 1: Figure S2).

## Discussion

We report the results of a high-density GWAS investigating genetic susceptibility to T2D-ESKD in 15,075 AAs. Top variants associated with T2D-ESKD were subsequently assessed for association with non-diabetic ESKD, and a meta-analysis was performed to test for their generalizability to common forms of ESKD. Eight independent associations in seven genetic loci displayed genome-wide significant association with T2D-ESKD in the baseline or *APOL1*-negative models, including *RND3/RBM43*, *SLITRK3*, *ENPP7*, *GNG7*, *APOL1*, *GRAMD3*, and *MGAT4C*. In addition to *APOL1*, two genome-wide significant loci were associated with all-cause ESKD, *EFNB2* and *GNG7*. Further, 10 genetic loci demonstrated nominal association with all-cause ESKD (*P* < 5 × 10^−6^), including *LPP*, *FSTL5*, *OPRK1/ATP6V1H*, *SYBU/KCNV1*, *ALK/YPEL5*, *MNX1-AS1/UBE3C*, *NUP98, LINC01075/LINC00448*, *TMCO5A*, and *SULF2/LINC01522*.

The most significant association with T2D-ESKD (OR = 0.77, *P* = 1.42 × 10^−10^) and all-cause ESKD (OR = 0.69, *P* = 1.96 × 10^−25^) in the baseline model was an intronic variant rs9622363 in the *APOL1* region associated with non-diabetic kidney disease in individuals with African ancestry. Conditioning on the *APOL1* G1 and G2 alleles dramatically diminishes its significance [6]. rs9622363 is reported to alter transcription factor (TF) binding motifs (Additional file 1: Table S6). In a recent study, rs9622363 and *APOL1* G1 alleles formed a haplotype that achieved the strongest association with CKD in Nigerians [19]. Unlike G1 or G2, the major allele in rs9622363 (G, EAF = 0.57) is associated with the risk of CKD. After excluding *APOL1* renal-risk-genotype carriers, association with rs9622363 was attenuated. This confirmed that rs9622363 and *APOL1* G1 and G2 alleles contribute to the same signal. Identification of rs9622363 in the baseline model may suggest misclassification of some cases as T2D-ESKD.

An intergenic variant (rs72858591) located between a GTPase protein gene *RND3* and *RBM43*, encoding RNA binding motif protein 43, revealed genome-wide significant association with T2D-ESKD. It is associated with TF binding motif changes and overlap with both promoter and enhancer regions (Additional file 1: Table S6). An independent intergenic variant (rs7560163, *r*^2^ = 0.01, YRI) in this region was previously associated with T2D in AAs [20]. In contrast, rs72858591 was not associated with T2D (*P* = 0.073) in the present study. This may suggest that two different sets of variations in this locus independently contribute to T2D and T2D-ESKD, a possible pleiotropic effect. Two independent variants (rs142563193 and rs142671759) that were genome-wide significantly associated with T2D-ESKD are located near *ENPP7*. These variants overlap with enhancer and promoter regions, DNase hypersensitive peaks, and/or TF binding motifs (Additional file 1: Table S6). The protein encoded by *ENPP7* is an intestinal alkaline sphingomyelin phosphodiesterase that converts sphingomyelin to ceramide and phosphocholine. *ENPP7* reportedly affects cholesterol absorption [21], and numerous studies suggest that high-density lipoprotein cholesterol levels are risk factors for CKD in patients with diabetes [22–24].

Two variants located in *GNG7* were associated with either T2D-ESKD (rs4807299; *P* = 3.21 × 10^−8^, baseline model) or all-cause ESKD (rs373971520; *P* = 2.17 × 10^−8^; *APOL1*-negative model). Rs4807299 is associated with TF binding motif changes and overlaps with both promoter and enhancer regions (Additional file 1: Table S6). *GNG7* encodes G Protein Subunit Gamma 7, involved in central nervous system function [25] and cancer risk [26, 27].

Since African Americans with diabetes and proteinuria often do not get a diagnostic kidney biopsy, their ESKD is typically presumed to have been caused by DKD. However, *APOL1*-associated non-diabetic kidney disease may be the true cause of kidney disease in many such patients. Analyses excluding *APOL1* renal-risk-genotype carriers created a more homogeneous case group and provided an opportunity to uncover the genetic architecture of T2D-ESKD that is independent of *APOL1* effect. In the *APOL1*-negative model, in addition to replicating *ENPP7* identified in the baseline model, two novel loci achieved genome-wide significant association with T2D-ESKD: *GRAMD3* (rs75029938; *P* = 2.02 × 10^−9^) and *MGAT4C* (rs17577888; *P* = 3.87 × 10^−8^). Functional annotation suggested that both variants are co-located with TF binding motifs. Rs75029938 may fall into enhancer and promoter regions, and rs17577888 was associated with transcript abundance of *FLVCR1* gene in peripheral blood monocytes (*P* = 6.41 × 10^−6^; Additional file 1: Table S6). Genetic variation in *GRAMD3* has been associated with adiposity in a multi-ethnic genome-wide meta-analysis [28]. Previous studies suggest that obesity is a major risk factor for DKD [29]. *MGAT4C* encodes Mannosyl (Alpha-1,3-)-Glycoprotein Beta-1,4-*N*-Acetylglucosaminyltransferase, Isozyme C, which participates in the transfer of *N*-acetylglucosamine (GlcNAc) to the core mannose residues of N-linked glycans. The potential involvement of *MGAT4C* in DKD requires further study.

This analysis included a cohort of AAs with non-diabetic ESKD to evaluate the generalizability of T2D-ESKD-associated loci in common forms of CKD. The meta-analysis combining cases with T2D-ESKD and non-diabetic ESKD identified two novel genome-wide significant loci associated with all-cause ESKD in addition to *APOL1*; rs77113398 near *EFNB2* (*P* = 9.84 × 10^−9^; baseline model) and rs373971520 in *GNG7* (2.17 × 10^−8^; *APOL1*-negative model). Rs77113398 overlaps with enhancer, promoter regions, and DNase peaks (Additional file 1: Table S6). Prior genome scans in AAs identified significant evidence for linkage to ESKD on chromosome 13q33 including the *EFNB2* region in both diabetic ESKD and non-diabetic ESKD [30, 31]. A follow-up study examined 28 tagging variants spanning 39 kilobases (kb) of the *EFNB2* coding region demonstrated nominal associations between two variants and all-cause ESKD [32]. However, these reported variants were not correlated with rs77113398. Ephrin-B2 (*EFNB2*) is expressed in the developing nephron; interactions between ephrin-B2 and its receptors play an important role in glomerular microvascular assembly [33]. In addition, ephrin-B2 reverse signaling protects against peritubular capillary rarefaction by regulating angiogenesis and vascular stability during kidney injury [34]. Ephrin-B1 also co-localizes with CD2-associated protein (CD2AP) and nephrin at the podocyte slit diaphragm and plays an important role in maintaining barrier function at the slit diaphragm [35]. Ephrin B4 receptor kinase transgenic mice develop glomerulopathy, manifested by fused afferent and efferent arterioles bypassing the glomeruli [36]. Thus, multiple lines of evidence support the potential *EFNB2* association with CKD, and it is the most promising causal gene underlying association of rs77113398.

This study has strengths and limitations. Although the multi-stage study design was well-powered including 15,075 AAs, it lacked replication of T2D-ESKD associations, particularly for rare variants. Furthermore, several genome-wide significant signals showed significantly different effect sizes across stages, which was likely attributable to sample size differences across stages, and a consequence of the “winner’s curse,” a phenomenon describing that the true genetic effect size is overestimated because of the initial positive finding. Future replication is needed to confirm these findings. There are few other existing collections with appropriate samples in AAs; this limited replication efforts. Moreover, it is difficult to exclude all individuals misclassified with DKD due to the frequent lack of kidney biopsies. Therefore, we carefully excluded samples with ESKD attributed to non-diabetic etiologies based on clinical phenotypes and subsequently excluded *APOL1* renal-risk-genotype carriers at high risk for non-diabetic ESKD. This should minimize misclassification.

## Conclusion

In conclusion, a GWAS was conducted in AAs with T2D-ESKD and seven genetic loci displayed genome-wide significant evidence of association, including in *RND3/RBM43*, *SLITRK3*, *ENPP7*, *GNG7*, *APOL1*, *GRAMD3*, and *MGAT4C*. Beyond *APOL1*, *EFNB2* and *GNG7* were also associated with non-diabetic ESKD and revealed genome-wide significant association with all-cause ESKD. Future investigations including genetic replication and experimental validation of these newly identified associations are required to assess their potential impacts on the biological processes leading to advanced DKD in populations with recent African ancestry.

## Methods

### Study participants

Study participants were recruited by the Wake Forest School of Medicine (WFSM; *N* = 8052), Family Investigation of Nephropathy and Diabetes (FIND; *N* = 926), Jackson Heart Study (JHS; *N* = 1912), Atherosclerosis Risk in Communities Study (ARIC; *N* = 2221), Coronary Artery Risk Development in Young Adults (CARDIA; *N* = 912), and Multi-Ethnic Study of Atherosclerosis (MESA; *N* = 1052). Analyses were approved by local institutional review boards, and all participants provided written informed consent. Cases were considered to have T2D-ESKD, including severe DKD, when diabetes was diagnosed ≥ 5 years prior to the onset of ESKD or with diabetic retinopathy to ensure adequate T2D duration, with renal replacement therapy, estimated glomerular filtration rate (eGFR) ≤ 30 ml/min/1.73 m^2^ (CKD4), or urine albumin to creatinine ratio (UACR) ≥ 300 mg/g (macroalbuminuria). Participants with CKD4 or macroalbuminuria (*N* = 138) were included as cases given their high risk of developing ESKD. T2D was diagnosed according to American Diabetes Association criteria with a fasting blood glucose ≥ 126 mg/dl, 2-h oral glucose tolerance test glucose ≥ 200 mg/dl, random glucose ≥ 200 mg/dl, use of diabetes medications, or physician-diagnosed diabetes. Cases with non-diabetic ESKD lacked diabetes (or had T2D for < 5 years) at the initiation of renal replacement therapy, and ESKD was attributed to chronic glomerular disease (e.g., focal segmental glomerulosclerosis), HIV-associated nephropathy, hypertension, or unknown cause. Patients with ESKD attributed to surgical or urologic causes, polycystic kidney disease, autoimmune disease, hepatitis, IgA nephropathy, membranous glomerulonephritis, membranoproliferative glomerulonephritis, or monogenic kidney diseases were excluded. Non-diabetic non-nephropathy controls included participants without diabetes or kidney disease (eGFR ≥ 60 ml/min/1.73 m^2^ and UACR < 30 mg/g). Subjects with T2D-lacking nephropathy had eGFR ≥ 60 ml/min/1.73 m^2^ and UACR < 30 mg/g.

### Sample preparation, genotyping, imputation, and quality control

The study participants were genome-wide genotyped using three different platforms: (1) 8704 samples recruited from WFSM, ARIC, CARDIA, JHS, MESA, and FIND were genotyped on the Affymetrix Genome-wide Human single nucleotide polymorphism (SNP) array 6.0 (Affy6.0); (2) 3133 samples recruited from WFSM were genotyped on the Affymetrix Axiom Biobank Genotyping Array (Axiom); and (3) 3238 samples recruited from WFSM were genotyped on the Illumina Multi-Ethnic Genotyping Array (MEGA). Quality control and imputation were performed separately by each genotyping platform as described below.

Variants that passed quality control (QC) were imputed to a combined haplotype reference panel including the 1000 Genomes phase 3 cosmopolitan reference panel (October 2014 version) [37] and a version of the African Genome Variation Project (AGVP) reference panel including 640 African ancestry haplotypes kindly provided by the African Partnership for Chronic Disease Research and Wellcome Trust Sanger Institute [38]. Pre-phasing was performed using SHAPEIT2 [39], and imputation was performed using IMPUTE2 [40]. Post-imputation QC was conducted to exclude variants with allele mismatch or with large frequency discrepancy (≥ 0.2) with the reference panel (0.2 × frequency in EUR + 0.8 × frequency in AFR) and imputation info score < 0.4. A subset of samples was directly genotyped for *APOL1* G1 and G2 variants using Sequenom (Sequenom, San Diego, CA). The concordance was 95% with imputed genotypes.

### Affy6.0 datasets

As described previously [41], 1513 T2D-ESKD cases, 5299 non-diabetic non-nephropathy controls, and 1892 T2D non-nephropathy controls from WFSM, FIND, JHS, ARIC, CARDIA, and MESA cohorts were genotyped using Affy6.0 (Table 1). In each study, standard QC measures were applied to exclude variants with call rate < 95%, minor allele frequency (MAF) < 0.01, or showing departure from Hardy-Weinberg Equilibrium (HWE) (*P* < 0.0001). Sample QC was performed to exclude subjects with call rates < 95%, DNA contamination, duplicates, or population outliers. Given that CARDIA, JHS, and MESA lacked cases with T2D-ESKD, samples from these studies were combined for imputation and association analyses along with WFSM, FIND, and ARIC.

### Axiom dataset

At WFSM, 1700 AA cases with T2D-ESKD, 770 AA controls without diabetes or nephropathy, and 663 AA controls with T2D who lacked nephropathy were genotyped on a customized Axiom genotyping array (Table 2). Detailed variant information, custom content design, including fine mapping of candidate regions, genotyping methods, and QC were previously reported [42]. In brief, this array included approximately 264K coding variants and insertions/deletions (indels), 70K loss-of-function variants, 2K pharmacogenomic variants, 23K eQTL markers, 246K multi-ethnic population-based genome-wide tag markers, and 115K custom content markers. Variants with call rates < 95%, departure from HWE (*P* < 0.0001), and monomorphic variants were excluded. A total of 724,530 variants were successfully called for downstream QC, imputation, and analyses. Sample QC was performed to exclude individuals with low call rate (< 95%), gender discordance, DNA contamination, duplication, or population outliers.

### MEGA dataset

At WFSM, 1910 non-diabetic ESKD cases, 219 T2D-ESKD cases, 201 controls with T2D lacking nephropathy, and 908 non-diabetic non-nephropathy controls were genotyped on the MEGA array (Table 2). This array was designed to improve fine-mapping and functional discovery by increasing variant coverage across multiple ethnicities. The array includes (1) backbone content containing highly informative variants for GWAS and exome analyses in ancestrally diverse populations and (2) custom content used to replicate or generalize index GWAS associations, augment GWAS tagging variants in priority regions, enhance exome content in priority regions, fine-map GWAS loci, identify functional regulatory variants, explore medically important variants, and identify novel variant loci in candidate pathways [43]. Genotyping was performed at WFSM. DNA from cases and controls were equally interleaved on 96-well plates to minimize artifactual errors during sample processing. A total of 48 samples sequenced as part of the 1000 Genomes Project [44] at the Coriell Institute for Medical Research were included in genotyping and had a concordance rate of 98.57%. Genotype calling was performed using GenomeStudio (Illumina, CA, USA). Variants with missing position, missing allele, allele mismatch, call rates < 95%, departure from HWE (*P* < 0.0001), frequency difference > 0.2 comparing with 1000 Genome Project phase 3 reference panel, and monomorphic variants were removed. Multiple probe sets were compared, and only the one with the highest call rate was kept. A total of 1,705,970 variants were successfully called for downstream QC, imputation, and analyses. Sample QC was performed to exclude individuals with low call rate (< 95%), gender discordance, DNA contamination, duplication, or population outliers. DNA swapping was identified and corrected.

### Statistical analysis

#### Discovery stage

We used a multi-stage study design to identify variants associated with T2D-ESKD and their potential role in all-cause ESKD. In the discovery stage, 3432 T2D-ESKD cases and 6977 non-diabetic non-nephropathy controls from all three datasets were included (Fig. 1). Association analysis was performed for each dataset using a logistic mixed model method implemented in the program GMMAT [45] under an additive genetic model. This method controls for population structure and cryptic relatedness through including a genetic relationship matrix (GRM) estimated from a set of high-quality autosomal variants as a random effect. The principal component analysis was performed using EIGENSOFT [46] for each genotyping platform. The first eigenvector (PC1) along with age and sex was used as covariates. A meta-analysis was performed in the three datasets using a fixed effect inverse variance weighting method implemented in METAL [47]. Suggestive associations for T2D-ESKD with *P* < 1 × 10^−5^, minor allele count (MAC) > 400, and heterogeneity *I*^2^ < 80 were selected for discrimination analysis.

#### Discrimination stage

To determine whether putative T2D-ESKD-associated loci in the discovery stage were driven by associations with T2D per se, a meta-analysis combining 2756 AAs with T2D-lacking nephropathy and 6977 non-diabetic non-nephropathy controls from the three datasets (Affy6.0, Axiom, and MEGA) was performed (stage 3, Fig. 1). Variants showing nominal association (*P* < 0.05) with T2D were excluded to remove T2D-associated variants.

#### Extension analysis of non-diabetic ESKD and meta-analysis of all-cause ESKD

Genetic variants showing suggestive association with T2D-ESKD (*P* < 1 × 10^−5^) but not associated with T2D were examined in a non-diabetic ESKD cohort including 1910 non-diabetic ESKD cases and 908 non-diabetic non-nephropathy controls from the WFSM-MEGA dataset for association with non-diabetic etiologies of kidney disease (stage 4). Variants showing nominal association (*P* < 0.05) were tested in a meta-analysis of all-cause ESKD using all T2D-ESKD, non-diabetic ESKD, and controls from the three datasets (*N* = 12,319, Affy6.0, Axiom, MEGA) (Fig. 1). This meta-analysis evaluated whether T2D-ESKD associations contributed to the risk of all-cause ESKD. We also looked up our top kidney disease-associated variants for putative association with T2D in AAs from the MEDIA consortium (*N* = 15,043 cases and 22,318 controls); SNPs with *P* < 0.05 after multiple comparison corrections were removed.

#### Exclusion of *APOL1* risk genotype carriers

The *APOL1* G1 and G2 alleles contribute to risk for non-diabetic kidney disease [6, 48]. To minimize misclassification of T2D-ESKD, a second analysis was performed excluding *APOL1* two-renal-risk-variant carriers and those with missing *APOL1* genotypes from T2D-ESKD cases and non-diabetic non-nephropathy controls (*APOL1*-negative model). This analysis reduced the heterogeneity of the population despite reducing sample size and having lower statistical power. Specifically, 308 T2D-ESKD cases and 630 controls from the Affy6.0 datasets, 323 T2D-ESKD cases, and 113 controls from the Axiom dataset, and 33 T2D-ESKD cases and 175 controls from the MEGA dataset were removed. In addition, we removed 891 of 1910 all-cause ESKD cases. This analysis may unmask the effects of other non-diabetic ESKD loci beyond *APOL1*. Individuals were considered *APOL1* renal-risk-variant carriers if they carried two G1 alleles (rs60910145 G allele, rs73885319 G allele), two G2 alleles (rs143830837, 6 base pair in-frame deletion), or were compound heterozygotes (one G1 and one G2 allele) [6].

#### Functional characterization

Proxies of genome-wide significant T2D-ESKD-associated variants (*r*^2^ ≥ 0.7 in 1000 Genomes AFR population) from the baseline and *APOL1*-negative models were selected using LDlink [49]. The lead variants and proxies were then queried for functional annotations from HaploReg [50], which included chromatin state and protein binding annotation from the Roadmap Epigenomics [51] and ENCODE projects [52], sequence conservation across mammals, the effect of variants on regulatory motifs, and gene expression from eQTL studies.

## Additional file

Additional file 1: **Figure S1.** QQ plot of GWAS results of T2D-ESKD vs. non-diabetic non-nephropathy controls under baseline model. **Figure S2.** *P* value comparisons between baseline, *APOL1*-negative, and *APOL1*-adjusted models. **Table S1.** Study description. **Table S2.** Genome-wide significant variants associated with T2D-ESKD at Stage 2 Meta-analysis. **Table S3.** Discrimination analysis in 2756 T2D-lacking nephropathy individuals and 6977 controls for genome-wide significant T2D-ESKD-associated variants in baseline mode. **Table S4.** Discrimination analysis in 2756 T2D-lacking nephropathy individuals and 6977 controls for genome-wide significant T2D-ESKD-associated variants in *APOL1*-negative model. **Table S5.** Results of *APOL1*-negative model for top associations identified in baseline model. **Table S6.** (Excel). Functional annotations of genome-wide significant T2D-ESKD variants and proxies in linkage disequilibrium (*r*^2^ > 0.7). (ZIP 138 kb)

## Abbreviations

AA: African Americans
AGVP: African Genome Variation Project
APOL1: Apolipoprotein L1
ARIC: Atherosclerosis Risk in Communities Study
CARDIA: Coronary Artery Risk Development in Young Adults
CKD: Chronic kidney disease
DKD: Diabetic kidney disease
EA: European Americans
EAF: Effect allele frequency
eGFR: Estimated glomerular filtration rate
ESKD: End-stage kidney disease
FIND: Family Investigation of Nephropathy and Diabetes
GRM: Genetic relationship matrix
GWAS: Genome-wide association analysis
MESA: Multi-Ethnic Study of Atherosclerosis
OR: Odds ratio
PC: Principal component
QC: Quality control
SNP: Single nucleotide polymorphism
T2D: Type 2 diabetes
T2D-ESKD: Type 2 diabetes-attributed kidney disease
UACR: Urine albumin to creatinine ratio
WFSM: Wake Forest School of Medicine
