# Supplementary material for: Genome-wide association study identifies novel loci for type 2 diabetes-attributed end-stage kidney disease in African Americans
Source: Hum Genomics. 2019 May 15;13:21. doi: 10.1186/s40246-019-0205-7 (PMC6521376; doi:10.1186/s40246-019-0205-7)
Supplement: Supplementary file 1 — Figure S1. QQ plot of GWAS results of T2D-ESKD vs. non-diabetic non-nephropathy controls under baseline model. Figure S2. P value comparisons between baseline, APOL1-negative, and APOL1-adjusted models. Table S1. Study description. Table S2. Genome-wide significant variants associated with T2D-ESKD at Stage 2 Meta-analysis. Table S3. Discrimination analysis in 2756 T2D-lacking nephropathy individuals and 6977 controls for genome-wide significant T2D-ESKD-associated variants in baseline mode. Table S4. Discrimination analysis in 2756 T2D-lacking nephropathy individuals and 6977 controls for genome-wide significant T2D-ESKD-associated variants in APOL1-negative model. Table S5. Results of APOL1-negative model for top associations identified in baseline model. Table S6. (Excel). Functional annotations of genome-wide significant T2D-ESKD variants and proxies in linkage disequilibrium (r2 > 0.7). (ZIP 138 kb) [file 40246_2019_205_MOESM1_ESM.zip › Guan_T2D-ESKD_GWAS_in_AAs_Supplementary_R2.docx]

**Supplementary Figure 1. QQ plot of GWAS results of T2D-ESKD vs. non-diabetic non-nephropathy controls under baseline model**

**
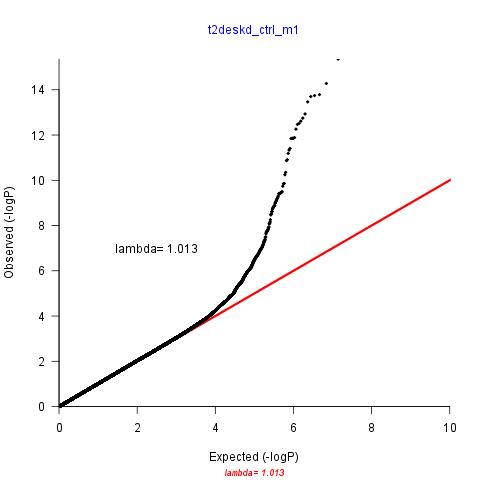
**

**Supplementary Figure 2. P value comparisons between baseline, *APOL1*-negative, and *APOL1*-adjusted models**

**
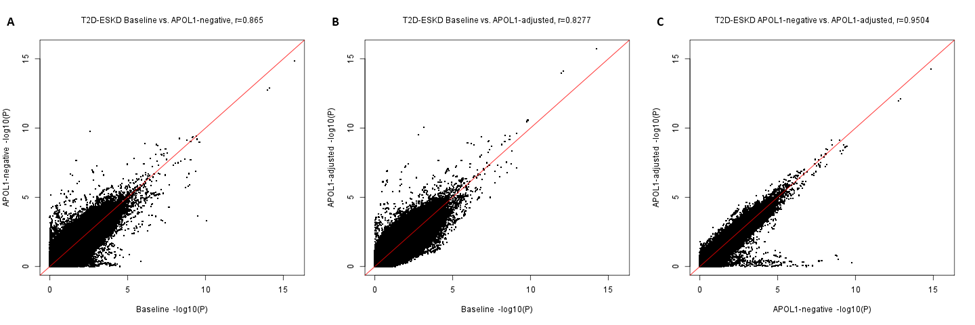
**

**Supplementary Table 1. Study description**

| **Study** | **Study name** | **Description** | **Reference** |
| --- | --- | --- | --- |
| ARIC | Atherosclerosis Risk in Communities Study | The ARIC study is a prospective population-based study of atherosclerosis and cardiovascular disease that included 15,792 participants (27% African Americans [AA]) aged 45-64 years at baseline visit between years 1987-1989 from four US communities. In this study, 96 AA subjects were included as type 2 diabetes-attributed end-stage kidney disease (T2D-ESKD) when type 2 diabetes (T2D) was diagnosed ≥5 years prior to the onset of ESKD or with diabetic retinopathy, with renal replacement therapy, estimated glomerular filtration rate (eGFR) <30 ml/min/1.73 m^2^ (CKD4) or urine albumin to creatinine ratio (UACR) ≥300 mg/g (macroalbuminuria) at the final visit. 1318 AA subjects with UACR<30 mg/g, eGFR ≥60 ml/min/m^2^ at all visits were included as non-diabetic, non-nephropathy controls. In addition, 807 AA subjects diagnosed as T2D with eGFR≥60 ml/min/1.73 m^2^ and UACR <30 mg/g at all visits were included as T2D-lacking nephropathy. | The ARIC Investigators (1989) The Atherosclerosis Risk in Communities (ARIC) Study: design and objectives. The ARIC investigators. Am J Epidemiol 129: 687-702. |
| CARDIA | Coronary Artery Risk Development in Young Adults Study | The CARDIA study is a prospective multi-center investigation of the natural history and etiology of cardiovascular disease that included 5,115 participants (52% African Americans) aged 18-30 years at baseline visit between years 1985-1986 from four US communities (Birmingham, AL; Chicago, IL; Minneapolis, MN and Oakland, CA). Follow-up examinations occurred at years 2, 5, 7, 10, 15, 20, 25, and 30. In this study, 747 AA subjects with UACR<30 mg/g, eGFR ≥60 ml/min/m2 were included as non-diabetic, non-nephropathy controls. In addition, 165 AA subjects diagnosed as T2D with eGFR≥60 ml/min/1.73 m2 and UACR <30 mg/g were included as T2D-lacking nephropathy. | Friedman GD, Cutter GR, Donahue RP, Hughes GH, Hulley SB, et al. (1988) CARDIA: study design, recruitment, and some characteristics of the examined subjects. J Clin Epidemiol 41: 1105-1116 |
| FIND | Family Investigation of Nephropathy and Diabetes | The Family Investigation of Nephropathy and Diabetes (FIND) is a multicenter study that is designed to find genes that contribute to the onset of diabetic nephropathy in four target, self-reported, heritage groups: European Americans, Mexican Americans, American Indians, and AAs. In this study, 627 AA subjects with T2D duration >5  years and/or diabetic retinopathy, with UACR >1 g/g or ESKD. 299 unrelated T2D-lacking nephropathy subjects had DM duration >9 years, UACR <30 mg/g, and serum creatinine <1.6 mg/dl (men) or <1.4 mg/dl (women) were also included. | J Diabetes Complications. 2005 Jan-Feb;19(1):1-9.  The Family Investigation of Nephropathy and Diabetes (FIND): design and methods.  Knowler WC1, Coresh J, Elston RC, Freedman BI, Iyengar SK, Kimmel PL, Olson JM, Plaetke R, Sedor JR, Seldin MF; Family Investigation of Nephropathy and Diabetes Research Group. |
| JHS | Jackson Heart Study | The JHS is a prospective population-based study to examine the risk factors of cardiovascular disease, T2D, obesity, chronic kidney disease and stroke among African Americans in the Jackson, Mississippi metropolitan area. A total of 5,306 self-identified African Americans were recruited from two cohorts of unrelated (aged 35-84 years) and nested family-based (aged ≥21 years) subjects during the baseline visit between years 2000-2004. In this study, 1569 AA subjects with UACR<30 mg/g, eGFR ≥60 ml/min/m2 were included as non-diabetic, non-nephropathy controls. In addition, 343 AA subjects diagnosed as T2D with eGFR≥60 ml/min/1.73 m2 and UACR <30 mg/g were included as T2D-lacking nephropathy. Subjects that also enrolled in ARIC were not included. | Taylor HA, Jr., Wilson JG, Jones DW, Sarpong DF, Srinivasan A, et al. (2005) Toward resolution of cardiovascular health disparities in African Americans: design and methods of the Jackson Heart Study. Ethn Dis 15: S6-4-17. |
| MESA | Multi-Ethnic Study of Atherosclerosis | The MESA is a study of the characteristics of subclinical cardiovascular disease (disease detected non-invasively before it has produced clinical signs and symptoms) and the risk factors that predict progression to clinically overt cardiovascular disease or progression of the subclinical disease. MESA researchers study a diverse, population-based sample of 6,814 asymptomatic men and women aged 45-84 who were enrolled between years 2000-2002. Approximately 38% of the recruited participants are white, 28% African-American, 22% Hispanic, and 12% Asian, predominantly of Chinese descent. Participants were recruited from six field centers (Wake Forest School of Medicine, Columbia University, Johns Hopkins University, University of Minnesota, Northwestern University, and University of California - Los Angeles). Key phenotypes included coronary calcification, ventricular mass and function, flow-mediated endothelial vasodilation, carotid intimal-medial wall thickness and presence of echogenic lucencies in the carotid artery, lower extremity vascular insufficiency, arterial wave forms, electrocardiographic (ECG) measures, standard coronary risk factors, socio-demographic factors, lifestyle factors, and psychosocial factors. DNA has been extracted and lymphocytes cryopreserved. Participants have been contacted every 9 to 12 months throughout the study to assess clinical morbidity and mortality. In this study, 774 AA subjects with UACR<30 mg/g, eGFR ≥60 ml/min/m^2^ were included as non-diabetic, non-nephropathy controls. In addition, 278 AA subjects diagnosed as T2D with eGFR≥60 ml/min/1.73 m^2^ and UACR <30 mg/g were included as T2D-lacking nephropathy. | Bild DE, Bluemke DA, Burke GL, Detrano R, Diez Roux AV, et al. (2002) Multi-ethnic study of atherosclerosis: objectives and design. Am J Epidemiol 156: 871-881. |
| WFSM | Wake Forest School of Medicine | The WFSM is a cross-sectional case-control study designed to examine the genetics of T2D and end-stage renal disease (ESRD) in African Americans. All subjects were recruited in North Carolina, South Carolina, Georgia, Tennessee, or Virginia. In this study, 2709 AA subjects were included as T2D-ESKD when T2D was diagnosed ≥5 years prior to the onset of ESKD or with diabetic retinopathy, with renal replacement therapy, eGFR <30 ml/min/1.73 m2 (CKD4) or UACR ≥300 mg/g (macroalbuminuria). 2569 AA subjects with UACR<30 mg/g, eGFR ≥60 ml/min/m^2^ were included as non-diabetic, non-nephropathy controls. 864 AA subjects diagnosed as T2D with eGFR≥60 ml/min/1.73 m^2^ and UACR <30 mg/g at all visits were included as T2D-lacking nephropathy. In addition, 1910 subjects with non-diabetic ESKD lacked diabetes (or had T2D for <5 years) at initiation of renal replacement therapy, and ESKD was attributed to chronic glomerular disease (e.g., focal segmental glomerulosclerosis), HIV-associated nephropathy, hypertension or unknown cause were included. Patients with ESKD attributed to surgical or urologic causes, polycystic kidney disease, autoimmune disease, hepatitis, IgA nephropathy, membranous glomerulonephritis, membranoproliferative glomerulonephritis, or monogenic kidney diseases were excluded.  WFSM includes samples from Arkansas African American metabolic cohort: Metabolic parameters were evaluated in nondiabetic individuals of African-American (n = 158, SI evaluated by FSIVGT in 157) heritage ascertained in Arkansas. 66 African American subjects from Arkansas underwent tolbutamide modified frequently sampled intravenous glucose tolerance test (FSIGT), whereas the remainder had an insulin modified FSIGT (Because tolbutamide became unavailable part way through the study). | McDonough CW, Palmer ND, Hicks PJ, Roh BH, An SS, et al. (2011) A genome-wide association study for diabetic nephropathy genes in African Americans. Kidney Int 79: 563-572.  Neeraj K. Sharma, Kurt A. Langberg, Ashis K. Mondal, Steven C. Elbein,† and Swapan K. Das. (2011) Type 2 Diabetes (T2D) Associated Polymorphisms Regulate Expression of Adjacent Transcripts in Transformed Lymphocytes, Adipose, and Muscle from Caucasian and African-American Subjects. J Clin Endocrinol Metab. 2011 Feb; 96(2): E394–E403. |

**Supplementary Table 2 (Excel). Genome-wide significant variants associated with T2D-ESKD at Stage 2 Meta-analysis**

**Supplementary Table 3. Discrimination analysis in 2756 T2D-lacking nephropathy individuals and 6977 controls for genome-wide significant T2D-ESKD associated variants in baseline model**

| **Variant** | **CHR** | **POS** | **Locus** | **Effect/Other allele** | **EAF** | **OR** | **P** |
| --- | --- | --- | --- | --- | --- | --- | --- |
| rs72858591 | 2 | 151711452 | *RBM43* | C/T | 0.091 | 1.12 | 0.073 |
| rs58627064 | 3 | 165051826 | *SLITRK3* | T/G | 0.058 | 1.14 | 0.083 |
| rs142563193 | 17 | 77667171 | *ENPP7* | A/G | 0.23 | 0.91 | 0.053 |
| rs142671759 | 17 | 77706698 | *ENPP7* | C/T | 0.021 | 1.29 | 0.070 |
| rs4807299 | 19 | 2570002 | *GNG7* | A/C | 0.048 | 1.14 | 0.15 |
| rs9622363 | 22 | 36656555 | *APOL1* | A/G | 0.47 | 0.96 | 0.25 |

Abbreviations: T2D, type 2 diabetes; ESKD, end-stage kidney disease; CHR, chromosome; POS, position; N, number; EAF, effect allele frequency; OR, odds ratio; P, p value.

**Supplementary Table 4. Discrimination analysis in 2756 T2D-lacking nephropathy individuals and 6977 controls for genome-wide significant T2D-ESKD associated variants in *APOL1*-negative model**

| **variant** | **CHR** | **POS** | **Locus** | **Effect/Other allele** | **EAF** | **OR** | **P** |
| --- | --- | --- | --- | --- | --- | --- | --- |
| rs75029938 | 5 | 125773333 | *GRAMD3* | T/C | 0.038 | 1.16 | 0.13 |
| rs17577888 | 12 | 87670213 | *MGAT4C* | T/G | 0.092 | 0.9 | 0.083 |
| rs142671759 | 17 | 77706698 | *ENPP7* | C/T | 0.021 | 1.29 | 0.07 |

Abbreviations: T2D, type 2 diabetes; ESKD, end-stage kidney disease; CHR, chromosome; POS, position; N, number; EAF, effect allele frequency; OR, odds ratio; P, p value.

**Supplementary Table 5. Results of *APOL1*-negative model for top associations identified in baseline model**

| **Variant** | **CHR** | **POS** | **EA** | **OA** | **EAF** | **N** | **OR** | **P** | **Phenotype** |
| --- | --- | --- | --- | --- | --- | --- | --- | --- | --- |
| rs75029938 | 5 | 125773333 | T | C | 0.042 | 10409 | 1.97 | 2.21E-12 | T2D-ESKD |
| rs73358292 | 10 | 114806988 | C | A | 0.067 | 10409 | 1.49 | 1.60E-07 | T2D-ESKD |
| rs17577888 | 12 | 87670213 | T | G | 0.089 | 10409 | 0.75 | 1.01E-05 | T2D-ESKD |
| rs142671759 | 17 | 77706698 | C | T | 0.023 | 10409 | 2.26 | 3.73E-09 | T2D-ESKD |
| rs12472637 | 2 | 30304514 | A | G | 0.33 | 12319 | 0.85 | 9.33E-06 | all-cause ESKD |
| rs76971802 | 3 | 188607071 | T | C | 0.089 | 12319 | 1.35 | 1.36E-06 | all-cause ESKD |
| rs6459733 | 7 | 156930550 | G | C | 0.39 | 12319 | 1.19 | 1.25E-06 | all-cause ESKD |
| rs4910809 | 11 | 3813850 | C | G | 0.023 | 12319 | 0.58 | 3.72E-06 | all-cause ESKD |
| rs219020 | 13 | 63013622 | C | T | 0.14 | 12319 | 1.28 | 1.54E-06 | all-cause ESKD |
| rs113452507 | 15 | 37954309 | C | G | 0.16 | 12319 | 1.25 | 5.07E-06 | all-cause ESKD |
| rs373971520 | 19 | 2568805 | D | I | 0.11 | 12319 | 1.37 | 1.72E-07 | all-cause ESKD |
| rs6094913 | 20 | 46561443 | G | A | 0.093 | 12319 | 0.77 | 3.18E-05 | all-cause ESKD |

Abbreviations: T2D, type 2 diabetes; ESKD, end-stage kidney disease; CHR, chromosome; POS, position; EA, effect allele; OA, other allele; N, number; EAF, effect allele frequency; OR, odds ratio; P, p value.

**Supplementary Table 6 (Excel). Functional annotations of genome-wide significant T2D-ESKD variants and proxies in linkage disequilibrium (r2>0.7).**
